# Engineering *Bacillus pumilus* alkaline serine protease to increase its low-temperature proteolytic activity by directed evolution

**DOI:** 10.1186/s12896-018-0451-0

**Published:** 2018-06-01

**Authors:** Hong-Yan Zhao, Hong Feng

**Affiliations:** 1 Key Laboratory for Bio-resources and Eco-environment of Ministry of Education, Sichuan Key Laboratory of Molecular Biology and Biotechnology, College of Life Sciences, Sichuan University, Chengdu, Sichuan 610064 People’s Republic of China; 2 Laboratory of Molecular Target Therapy of Cancer, Institute of Basic Medical Sciences, Hubei University of Medicine, Shiyan, 442000 Hubei People’s Republic of China

**Keywords:** Serine protease, Cold activity, Thermostability, Enzyme kinetics, Directed evolution

## Abstract

**Background:**

Mesophilic alkaline serine proteases from various bacteria have been commercially applied in a range of industries owing to their high catalytic efficiency and wide substrate specificity. However, these proteases have an optimal catalytic temperature of approximately 50 °C, and their activity decreases significantly at low temperature. Therefore, to enhance their cold activity, it is necessary to improve the catalytic performance of these proteases at low temperature. The alkaline serine protease (DHAP) from *Bacillus pumilus* BA06 is a typical mesophilic enzyme, which has demonstrated great potential in various industrial applications. Here we attempted to improve the cold activity of DHAP via directed evolution.

**Results:**

Seven variants (P9S, A1G/K27Q, A38V, A116T, T162I, S182R, and T243S) of DHAP from *B. pumilus* were obtained via directed evolution. The results showed that all of the variants had increased proteolytic activity at 15 °C towards both the casein and synthetic peptide substrates. With the exception of variant T243S, the thermostability of these variants did not decrease in comparison with the wild-type enzyme. Kinetic analysis indicated that the increase in catalytic efficiency was largely attributed to the increase in turnover number (*k*_cat_). Furthermore, the combined variants generated by site-directed mutagenesis showed a further increase in specific caseinolytic activity and the *k*_cat_ value for hydrolysis of the synthetic peptide. The combined variants of P9S/K27Q and P9S/T162I exhibited an approximate 5-fold increase in caseinolytic activity at 15 °C and almost no loss of thermostability. Finally, the possible mechanism responsible for the change in catalytic properties for these variants was interpreted based on structural modeling.

**Conclusions:**

Directed evolution and site-directed mutagenesis were combined to engineer variants of the DHAP from *B. pumilus*. All of the variants exhibited an increase in hydrolytic efficiency at low temperature towards both of the substrates, casein and synthetic peptide, without any loss of thermostability compared with the wild-type. These data suggest that engineering low-temperature activity for a bacterial protease is not always associated with the loss of thermostability. Furthermore, our findings demonstrate that enhanced cold activity and thermostability could be integrated into a single variant.

**Electronic supplementary material:**

The online version of this article (10.1186/s12896-018-0451-0) contains supplementary material, which is available to authorized users.

## Background

The alkaline serine proteases from various bacteria are commercially important enzymes that are widely used in a variety of industries such as laundry, leather, and silk processing [1, 2]. Therefore, bacterial serine proteases have been extensively characterized in terms of their catalytic mechanisms [3, 4]. The proteases from mesophilic bacteria usually exhibit higher catalytic efficiency at 45–50 °C, and their activity decreases rapidly at low temperature. Recently reported improvements in the cold activity of bacterial serine proteases have attracted great attention because such enzymes are commercially important in various biotechnological processes [5, 6]. Although cold-adapted proteases with higher catalytic efficiency at low temperature have been purified from psychrophilic bacteria, these enzymes are commonly thermosensitive [7, 8]. Alternatively, it has been proposed that protein engineering approaches could be employed to improve the cold activity of mesophilic serine proteases [9–11]. For example, the P3C9 mutant of the mesophilic serine-like protease (SSII) from *Bacillus sphaericus* was constructed through molecular evolution and exhibited a 9.6-fold increase in catalytic efficiency (*k*_cat_/*K*_m_) compared with the wild-type (wt) enzyme [12].

Several alkaline serine proteases from *Bacillus pumilus* have been purified and characterized biochemically [13–17]. They were found to belong to the subtilisin family of enzymes and have been assigned as endopeptidase Q in the MEROPS database [18]. Endopeptidase Q shows desirable properties, such as high catalytic efficiency and good thermostability, suggesting the potential of this enzyme family in various industrial applications [19]. However, only limited examples of protein engineering have been applied to this group of proteases [20]. Previously, an alkaline serine protease from *B. pumilus* BA06, designated dehairing alkaline protease (DHAP), was purified and characterized [15, 21] and has shown great potential in leather processing [22]. However, wt DHAP undergoes caseinolytic activity at an optimal temperature 55 °C [15], which may not be suitable for practical operation in leather processing. Therefore, there is a need to improve the catalytic activity of this protease at lower temperatures, especially for industrial applications. Previously, we have engineered several DHAP variants by site-directed mutagenesis based on homologous sequence alignments and structural modeling and demonstrated improvements in their catalytic performance compared with the wt enzyme [23, 24].

In this study, a random mutant library of DHAP was constructed and extensively screened, focusing particularly on cold activity. Several variants exhibited a more than 5-fold increase in caseinolytic activity and a more than 2-fold increase in catalytic efficiency towards the synthetic peptide (AAPF-*p*N) at 15 °C. The adaptation mechanism of the DHAP variants to low temperature was also discussed based on the structural modeling of DHAP.

## Methods

### Materials

The synthetic peptide substrate *N*-succinyl-*L*-Ala-Ala-Pro-Phe-*p*-nitroanilide (AAPF-*p*N) and casein were purchased from Sigma-Aldrich (St Louis, MO, USA). Other chemicals used in this study were of reagent grade quality. Restriction enzymes, Tma endo V and dITP, were purchased from Thermo Scientific (Waltham, MA, USA); Taq™ DNA polymerase and PrimeSTAR® HS DNA polymerase were purchased from TaKaRa Biotech. Co. (Dalian, China).

*Escherichia coli* JM109 was used to construct the mutation library; *Bacillus subtilis* WB600 served as a host in which to express DHAP and its variants [25].

### Methods

#### Construction of the mutation library

The mutation library of DHAP was constructed following the protocol described previously [26]. Briefly, the error PCR was performed in a 100-μL mixture containing 5 U Taq DNA polymerase, 50 μM dNTPs, 150 μM dITP, 400 nM primers (DEAP-Hin.F/DEAP-Bam.R, Additional file 1: Table S1), and 10 ng template DNA of pAPN8B, which harbored the full-length sequence encoding the alkaline serine protease DHAP as well as its promoter and terminal sequences [24]. The PCR conditions were set as: predenaturation for 2 min at 94 °C; then 30 cycles of denaturation for 30 s at 94 °C, annealing for 30 s at 52 °C, and extension for 1 min at 72 °C; followed by a final extension for 10 min at 72 °C. The PCR products were treated with *Dpn*I to remove the template DNA, and were then digested with Tma endo V. The DNA fragments within the range of 50 to 100 bp were recovered on a 2% agarose gel. About 100 ng of DNA fragments were used as template to assemble a DNA product by PCR in the absence of primers. This reaction was performed in a 100-μL mixture by predenaturation for 2 min at 94 °C; then 42 cycles of denaturation for 30 s at 94 °C, annealing for 30 s at 42 °C, and extension at 72 °C for 10 s plus 2 s per cycle; followed by a final extension for 10 min at 72 °C. The resulting assembled products were used as template DNA to amply the full-length mutated genes by PCR using primers DEAP-Hin.F and DEAP-Bam.R. This reaction was performed in a 100-μL mixture containing 2.5 U Taq DNA polymerase, 200 μM dNTPs, and 400 μM of each primer. The PCR conditions involved pre-denaturation for 2 min at 94 °C; then 25 cycles of denaturation for 30 s at 94 °C, annealing for 30 s at 52 °C, extension for 1 min at 72 °C; followed by a final extension for 10 min at 72 °C.

The DNA products were purified with the Omega Gel Recover Kit (Bio-Tek, Inc. Norcross, GA, USA) and digested with *Bam*H I and *Hin*d III, and then ligated with pAPN8B [23] digested with the same restriction endonucleases. The ligation products were transformed into *E. coli* JM109. Finally, more than 100,000 independent transformants were harvested from the LB agar plates and used to isolate the plasmid DNAs, which were stored at -20 °C as the mutation library.

To evaluate the mutation frequency, 21 independent transformants were randomly selected for DNA sequencing.

#### Screening of the mutation library

Plasmid DNAs from the mutation library were transformed into *B. subtilis* WB600 [27], then spread onto LB agar plates supplemented with 1% milk powder, 25 μg/mL kanamycin (Kan), and 20 μg/mL chloramphenicol (Cm). Clones with larger hydrolytic diameters were randomly picked and transferred into each of the wells of 96-well plates containing 1 mL of LB broth plus 1% milk powder. The plates were incubated for 48 h at 37 °C with gentle shaking. Then, the supernatant was collected by centrifugation at 4000 rpm for 10 min and 20 μL of supernatant was transferred into a 96-well plate. To each well, 100 μL of 1% milk powder dissolved in boric-glycerin buffer (pH 9.6) was added to serve as substrate. The hydrolytic reaction was performed at 50 °C for 10 min and 15 °C for 30 min, and the OD_590_ was monitored on a μQuant Microplate Spectrophotometer (BioTek Instruments, Winooski, VT, USA). In addition, another set of the supernatant samples was pre-treated at 60 °C for 15 min and the survival activity was measured at 50 °C as described above. Through the primary screening described above, 1500 independent *B. subtilis* transformants were screened out of approximately 30,000 clones.

The 1500 transformants were then further screened by inoculation into 3 mL of LB plus 1% milk powder in 15-cm diameter test tubes and incubation at 37 °C for 48 h with shaking. The supernatant was also collected by centrifugation at 4000 rpm, and then subjected to an activity assay as described above. Finally, 36 mutant clones were obtained.

The plasmid DNAs were extracted from the *B. subtilis* cells, and then transformed into *E. coli* JM109. Finally, 36 mutated genes were obtained by DNA sequencing.

#### Expression and purification of the proteases

The 36 mutant clones were transformed into *B. subtilis* WB600 again, then each clone was inoculated to 3 mL of LB (containing 20 μg/mL Cm and 25 μg/mL Kan) and incubated overnight at 37 °C with shaking at 180 rpm. Then, 1 mL of each culture was transferred into 1 L of LB broth supplemented with 1% milk powder, 20 μg/mL Cm, and 25 μg/mL Kan, and incubated for 48 h at 37 °C with shaking at 200 rpm. The supernatant was collected by centrifugation at 4600 *g* for 10 min at 4 °C. Finally, the recombinant DHAP and its variants were purified and quantified as previously described [23]. Finally, the recombinant proteases were adjusted to a concentration of 0.5 μg/μL in storage buffer (25 mM Tris-HCl, 2 mM CaCl_2_, 50% glycerol, pH 8.0).

#### Construction of combined variants

The three amino acid substitutions, P9S, K27Q, and V162I, were combined to generate new DHAP variants following the QuickChange protocol (Stratagene Co., La Jolla, CA, USA). The primers used are shown in Additional file 1: Table S1. The mutagenesis PCR was performed in a 25-μL mixture containing ~ 100 ng mutated plasmid DNA, 2.5 U PrimeSTAR® DNA polymerase, 400 nM each primer, and 200 μM dNTPs. The PCR conditions were as follows: pre-denaturation for 4 min at 94 °C; then 25 cycles of denaturation for 30 s at 94 °C, annealing for 30 s at 52 °C, and extension for 6.5 min at 72 °C; followed by a final extension for 10 min at 72 °C. Then, 5 U of *Dpn* I was directly added into the PCR mixture to digest the template DNA at 37 °C for 4 h. Following transformation of the PCR products into *E. coli* JM109, the mutants were confirmed by DNA sequencing. Finally, expression and purification of the new DHAP variants were performed as described above.

#### Enzyme activity assay

For the purified DHAP and its variants, caseinolytic activity was assayed according to a standard procedure [21]. Briefly, a 400-μL reaction mixture was set up by the addition of 100 μL of boric acid–NaOH buffer (pH 9.6), 200 μL 1% casein, 2 μL 200 mM CaCl_2_, and 96 μL H_2_O. The hydrolytic reaction was started by the addition of 2 μL of enzyme solution, and incubated at 50 °C or the indicated temperature for exactly 10 min. The reaction was terminated by the addition of 400 μL of 40% trichloroacetic acid. After standing at room temperature for 15 min, the mixture was centrifuged at 12,000 rpm for 10 min. Then, 0.2 mL of supernatant was mixed with 1 mL of 0.4 M Na_2_CO_3_ and 0.2 mL of Folin reagent. The mixture was incubated for 20 min at 50 °C, then the absorbance was measured at 680 nm. One caseinolytic unit was defined as the amount of enzyme that produced 1 μg of tyrosine per min.

Hydrolysis of AAPF-*p*N was performed in 20 mM Tris-HCl buffer (pH 8.0) containing 2 mM CaCl_2_ with the indicated amounts of substrate and enzyme. The hydrolytic reaction was started by the addition of substrate, and the release of *p*-nitroanilide was monitored online at 410 nm using a UV2450 spectrophotometer (Shimadzu, Kyoto, Japan). The concentration of *p*-nitroanilide was calculated using *ε*_410_ = 8880 M^− 1^·cm^− 1^ as the molar extinction coefficient.

For estimation of the kinetic parameters, the initial velocity was estimated using a range of substrate concentrations (0.05–1.2 mM) at the indicated temperature. The obtained values for the initial velocity for a particular substrate concentration were used to fit to the Michaelis–Menten equation by nonlinear regression using the software Sigma Plot v12 (http://www.sigmaplot.com).

All of the above experiments were carried out in triplicate.

#### Thermostability

To evaluate the thermostability of wt DHAP and its variants, an appropriate amount of enzyme was diluted in 25 mM Tris-HCl buffer (pH 8.0) and incubated in a water bath at 60 °C for different time periods. At the indicated time points, an enzyme sample was withdrawn and immediately immersed in ice. The residual activity was assayed as described above using casein as substrate. The experiments were repeated in triplicate.

#### Homology modeling

Homology structural modeling of DHAP was performed on the Swiss–Model Server [28] using the subtilisin protease (PDB number: 1scjA) [29] as template. The structural models were viewed and analyzed using the software SPDB V4.0 [30].

## Results

### Construction and evaluation of a mutation library

Using a protocol involving dITP for error-prone PCR and DNA shuffling [26], a mutation library for the alkaline protease DHAP was constructed that contained more than 100,000 independent clones. To evaluate the mutation frequency, 21 clones were randomly picked for DNA sequencing. The results showed that 20 clones were mutated and contained at least one mutation site. Overall, the mutation frequency is about three mutations per kilobase of DNA. Twelve clones contained one to three nucleotide mutations, six clones contained four to six nucleotide mutations, and two clones with more than seven mutation sites. The specific mutation patterns are summarized in Table 1. Similar to previous results [26], the conversion of C to T or A to G was a dominant mutation, accounting for more than 87% of mutations. By contrast, transversion events occurred much less frequently.

**Table 1:** Nucleotide substitutions in the DHAP gene in the mutation library

Mutation types	Nucleotide change	Number	Percentage (%)
Transitions	A → G	11	20.37
	G → A	9	16.67
	C → T	16	29.63
	T → C	11	20.37
	Total	47	87.04
Transversions	G → C	1	1.85
	C → G	2	3.70
	C → A	3	5.56
	A → T	1	1.85
	Total	7	12.96

### Screening of the mutation library

Approximately half of the clones from the library exhibited milk-hydrolytic activity on milk-containing agar plates after transformation into *B. subtilis* WB600. Approximately 30,000 transformants with caseinolytic activity were picked and transferred into 96-deep-well plates for screening of low-temperature activity and thermostability. Through the first round of screening, about 1500 clones with improved low-temperature hydrolytic activity or thermostability were obtained. Following second-round screening of these 1500 clones, 36 clones were confirmed to exhibit activity at low temperature.

By DNA sequencing, the mutated genes of these 36 clones were obtained, among which 24 clones contained single amino acid site mutations, eight clones contained two amino acid site mutations, and four clones contained three mutation sites or more (Additional file 2: Table S2).

### Temperature-dependent caseinolytic activity of the selected variants

To characterize the catalytic properties of the clones, the 36 clones were transformed into *B. subtilis* WB600 again, and the recombinant proteins were purified and quantified. Additional file 3: Figure S1 shows the results of SDS–PAGE analysis of the wt and selected variants. The caseinolytic activity was primarily assayed at both 15 °C and 50 °C for the 36 variants and the wt of DHAP. The results are summarized in Additional file 2: Table S2 and demonstrated that the low-temperature activity of many of the variants was increased compared with the wt.

**Fig. 1:**
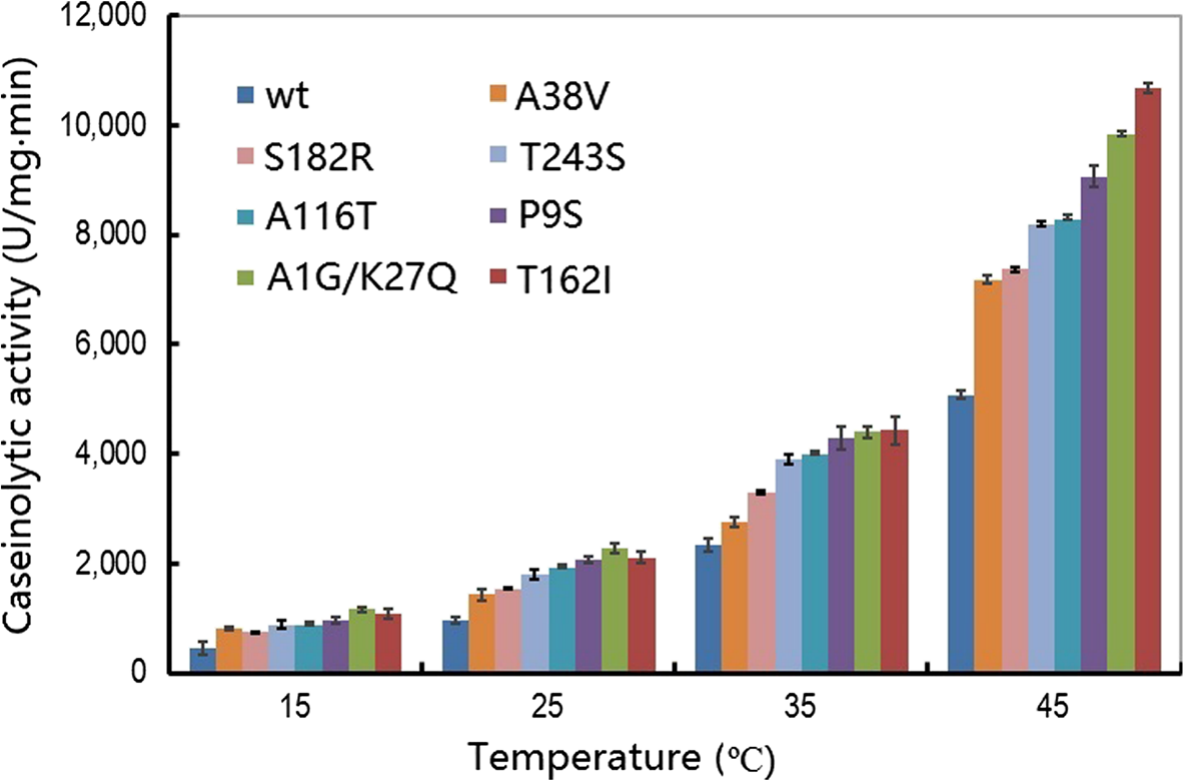
Temperature-dependent caseinolytic activity of the wild-type and variants of the alkaline serine protease DHAP. An activity assay was performed in a 400 μL reaction mixture (borate/NaOH buffer, pH 9.6, 2 mM CaCl_2_) containing 1% casein as substrate and 0.2 μg enzyme for 10 min. The data are expressed as the average value from three independent experiments with the experimental deviation shown

Subsequently, seven variants were selected for further assays. First, the specific caseinolytic activity was evaluated at various temperatures. Figure 1 shows that all of the seven variants achieved enhanced caseinolytic activity at different temperatures compared with the wt. In particular, the caseinolytic activity of two variants (A1G/K27Q and T162I) was increased by 2.62- and 2.41-fold at 15 °C, respectively, compared with the wt. However, the fold change was reduced at higher temperatures.

**Fig. 2:**
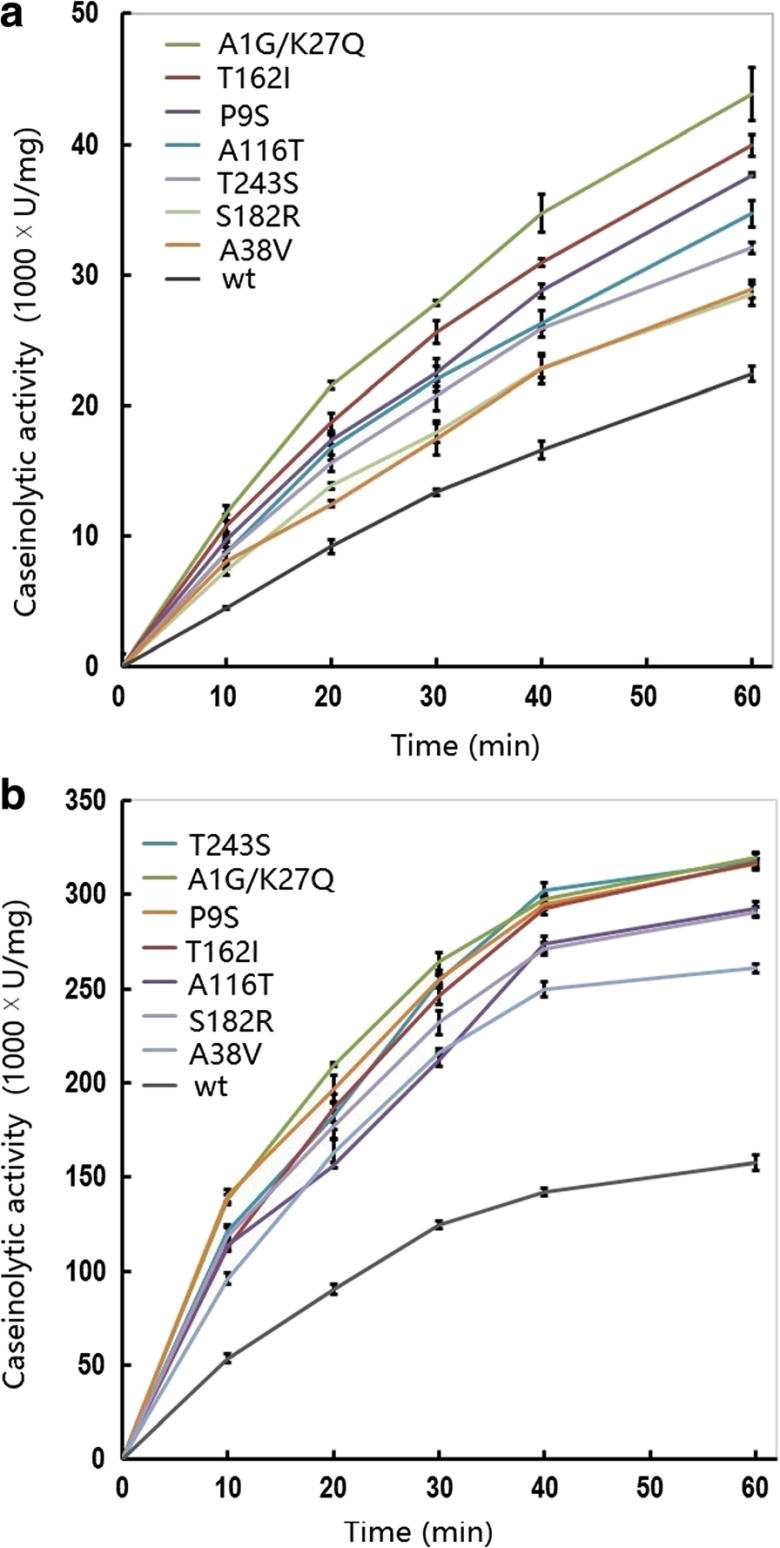
Caseinolytic progress curve of the wild-type and variants of the alkaline serine protease DHAP. Data are shown for reactions performed at 15 °C (**a**) and 50 °C (**b**). The activity assay was performed in a 400 μL reaction mixture (borate/NaOH buffer, pH 9.6, 2 mM CaCl_2_) containing 1% casein as substrate and 0.2 μg enzyme. At the indicated time-points, 400 μL of reaction mixture was withdrawn and the catalytic reaction was immediately stopped by the addition of 10% trichloride acetate. The data are expressed as the average value from three independent experiments with the experimental deviation shown

The caseinolytic progression over time was also monitored at 15 °C and 50 °C for these variants (Fig. 2a, b). Overall, the progress curves of caseinolytic activity obeyed a first-order exponential increase. Therefore, the slope of the curve in the linear phase could be used to calculate the reaction velocity and the catalytic rate was obviously enhanced for the variants compared with the wt.

### Catalytic properties of the combined variants

**Fig. 3:**
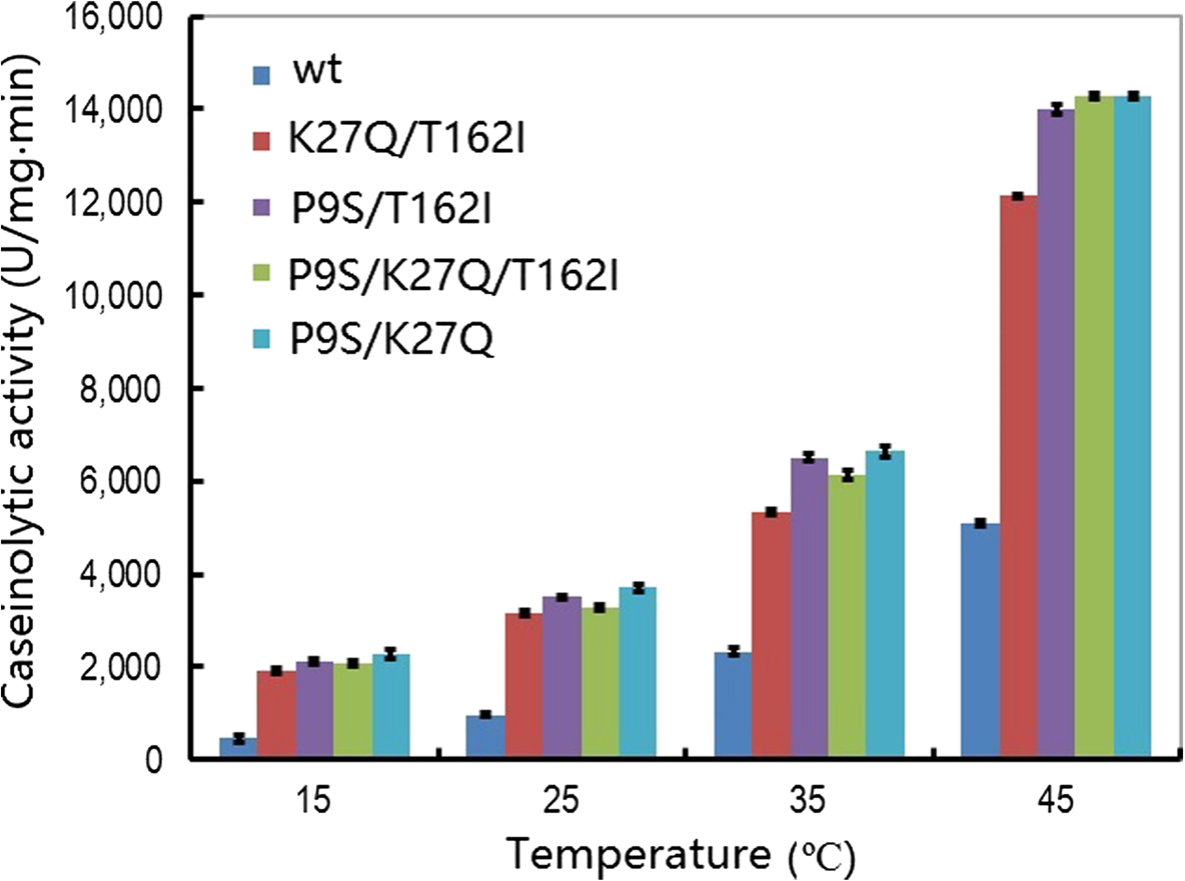
Temperature-dependent caseinolytic activity of the wild-type and combined variants of the alkaline serine protease DHAP. The activity assay was performed in a 400 μL reaction mixture (borate/NaOH buffer, pH 9.6, 2 mM CaCl_2_) containing 1% casein as substrate and 0.2 μg of enzyme for 10 min. The data are expressed as the average value from three independent experiments with the error bars shown

**Fig. 4:**
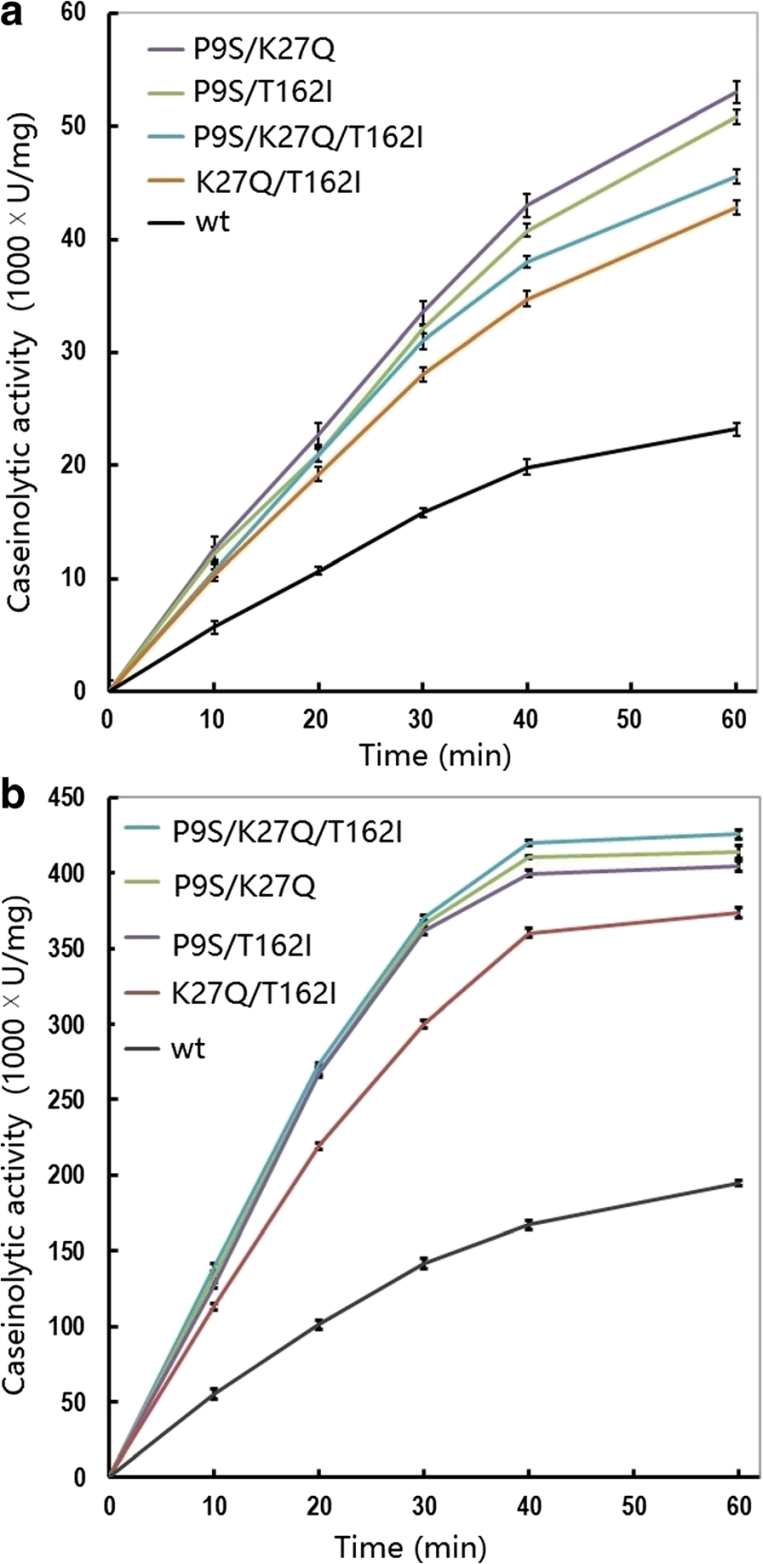
Caseinolytic progress curve of the wild-type and combined variants of the alkaline serine protease DHAP. Data are shown for reactions performed at 15 °C (**a**) and 50 °C (**b**). The activity assay was performed in a 400 μL reaction mixture (borate/NaOH buffer, pH 9.6, 2 mM CaCl_2_) containing 1% casein as substrate and 0.2 μg of enzyme. At the indicated time-points, 400 μL of reaction mixture was withdrawn and the catalytic reaction was immediately stopped by the addition of 10% trichloride acetate. The data are expressed as the average value from three independent experiments with the experimental deviation shown

Based on the above results, four new variants were constructed by the combination of three amino acid substitutions: P9S, K27Q, and T162I. In these variants, caseinolytic activity was further improved compared with the original substitutions at various temperatures (Fig. 3). For example, the caseinolytic activity of the mutant (P9S/K27Q, P9S/T162I, P9S/K27Q/T162I) was increased by about 5-fold and 2.8-fold compared with the wt at 15 °C and 50 °C, respectively. The progress curves of caseinolytic activity for the four combined variants are shown in Fig. 4a and b. The proteolytic rate was higher for all of the variants compared with the wt.

### Hydrolytic kinetics towards AAPF-*p*N

The kinetic parameters for AAPF-*p*N were calculated for these variants (Table 2). All of the variants showed increased catalytic efficiency (*k*_cat_/*K*_m_) to varying extents at 15 °C. It was notable that all of the variants showed an increased *K*_m_, except for the A38V variant whose *K*_m_ value was reduced compared with wt. These results indicated that the increased catalytic efficiency (*k*_cat_/*K*_m_) of these variants was largely ascribed to an increase in the turnover number (*k*_cat_). For two of the variants (P9S and A1G/K27Q), the *k*_cat_ value was increased by > 3.5-fold more than that of the wt. Furthermore, the catalytic efficiency at 50 °C was also enhanced for all of the variants, with the exception of the A1G/K27Q variant, but showed a lower fold-change compared with that at 15 °C.

**Table 2:** Kinetic parameters of the alkaline serine protease DHAP and its variants at different temperatures^a^

Variants	15 °C				50 °C			
	*K*_m_ (mM)	*k*_cat_	*k*_cat_/*K*_m_ (*s*^− 1^·M^− 1^)	Fold (◊)	*K*_m_ (mM)	*k*_cat_	*k*_cat_/*K*_m_ (*s*^− 1^·M^− 1^)	Fold (◊)
		(*s*^−1^)				(*s*^−1^)		
wt	0.16 ± 0.02	102 ± 03	0.64E × 10^6^	1.00	0.23 ± 0.018	609 ± 14	2.65E × 10^6^	1.00
P9S	0.41 ± 0.09	362 ± 43	0.88E × 10^6^	1.37	0.43 ± 0.045	829 ± 81	1.97E × 10^6^	0.74
A1G/K27Q	0.20 ± 0.04	373 ± 27	1.87E × 10^6^	2.92	0.36 ± 0.081	1719 ± 161	4.78E × 10^6^	1.83
A38V	0.13 ± 0.02	176 ± 11	1.35E × 10^6^	2.11	0.20 ± 0.096	952 ± 123	4.76E × 10^6^	1.79
A116T	0.22 ± 0.05	262 ± 22	1.19E × 10^6^	1.86	0.25 ± 0.040	778 ± 71	3.11E × 10^6^	1.17
T162I	0.17 ± 0.03	300 ± 20	1.76E × 10^6^	2.75	0.26 ± 0.026	1149 ± 53	4.41E × 10^6^	1.66
S182R	0.19 ± 0.03	259 ± 18	1.36E × 10^6^	2.13	0.23 ± 0.50	897 ± 102	3.90E × 10^6^	1.47
T351S	0.31 ± 0.05	283 ± 24	0.91E × 10^6^	1.42	0.25 ± 0.053	989 ± 97	3.96E × 10^6^	1.49
P9S/T162I	0.30 ± 0.013	450 ± 10.2	1.50E × 10^6^	2.34	0.26 ± 0.009	1169 ± 22	4.50E × 10^6^	1.70
P9S/K27Q	0.35 ± 0.035	645 ± 28.5	1.84E × 10^6^	2.88	0.32 ± 0.015	1555 ± 40	4.86E × 10^6^	1.83
K27Q/T162I	0.21 ± 0.013	288 ± 5.9	1.37E × 10^6^	2.14	0.25 ± 0.018	1092 ± 22	4.37E × 10^6^	1.65
P9S/K27Q/T162I	0.34 ± 0.016	579 ± 19.9	1.70E × 10^6^	2.65	0.34 ± 0.016	1596 ± 47	4.69E × 10^6^	1.77

^a^The hydrolytic reaction was performed in 20 mM Tris-HCl buffer (pH 8.0) containing 2 mM CaCl_2_ with 0.05–1.2 mM AAPF-*p*N as substrate and 50 ng (at 15 °C) or 25 ng (at 50 °C) of enzyme

For the combined variants, the turnover number (*k*_cat_) was further enhanced compared with the wt at 15 °C. The *k*_cat_ value of variants (P9S/T162I, P9S/K27Q, and P9S/K27Q/T162I) was significantly increased compared with the original substitutions (P9S, K27Q, and T162I). These results suggest that the individual substitutions may positively cooperate to improve the turnover number (*k*_cat_) during the catalytic process. However, the catalytic efficiency (*k*_cat_/*K*_m_) of these variants was not improved significantly compared with the original variants owing to an increase in *K*_m_ values.

### Thermostability

**Fig. 5:**
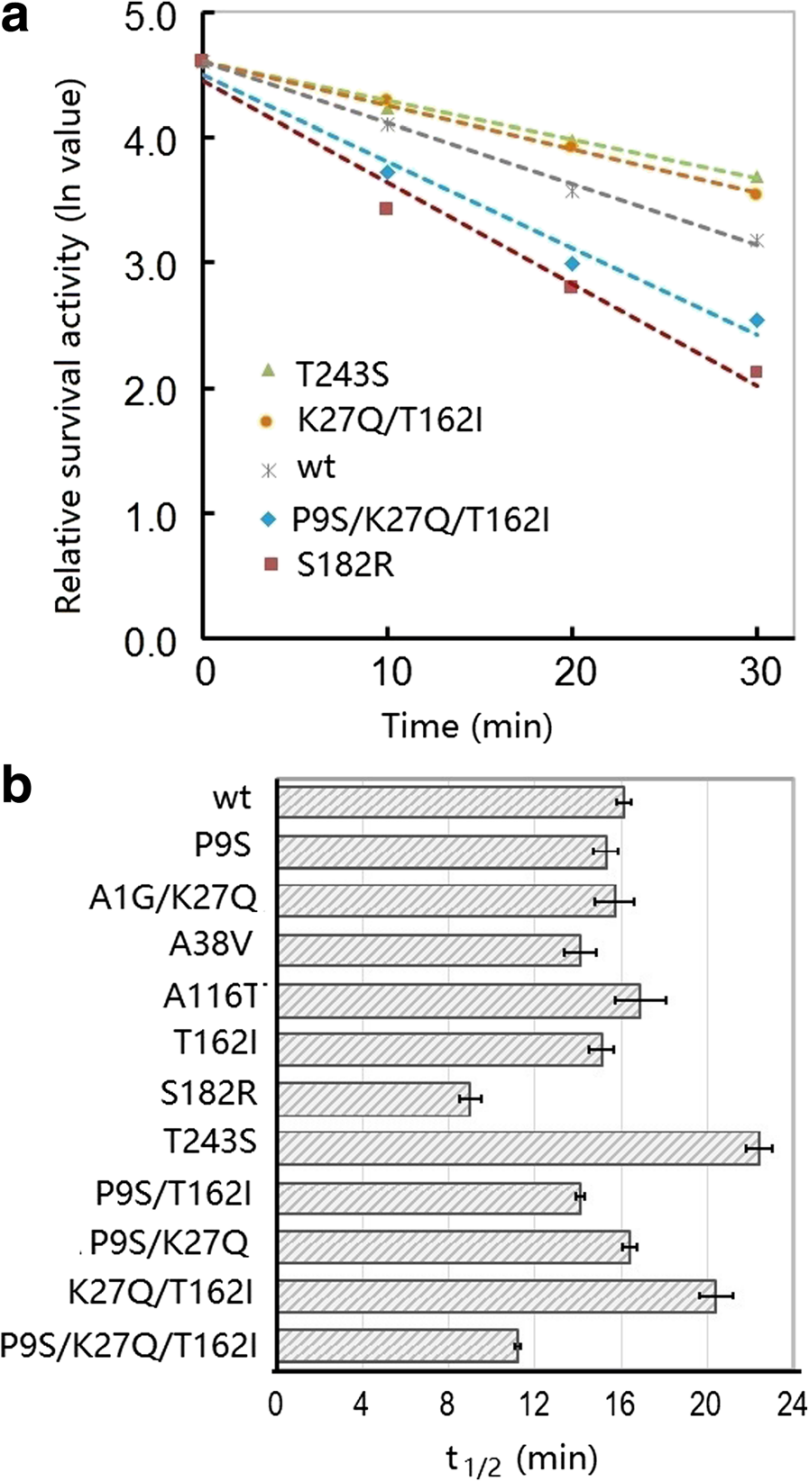
Thermal inactivation profiles (**a**) and half-lives (t_1/2_; **b**) of the wild-type and variants of DHAP. The enzyme (0.5 μg) was diluted in borate/NaOH buffer (pH 9.6) and incubated at 60 °C for various times. Then, the survival activity was assayed in a 400 μL reaction mixture (borate/NaOH buffer, pH 9.6, 2 mM CaCl_2_) containing 1% casein as substrate for 10 min. The relative activity was normalized to the initial activity (100%). The data are expressed as the average value from three independent experiments

Thermostability is an important parameter for industrial enzymes. Therefore, thermostability was evaluated for the variants in this study. Figure 5a shows the thermal inactivation curve of the wt and selected variants. The slope of two variants (T243S and K27Q/T162I) was slower than that of the wt, indicating that their thermostability was improved. By contrast, the thermostability of two variants (S182R and P9S/K27Q/T162I) was reduced compared with the wt. The other variants exhibited similar *t*_2/1_ values (time required for loss of half the catalytic activity) to the wt (Fig. 5b).

## Discussion

**Fig. 6:**
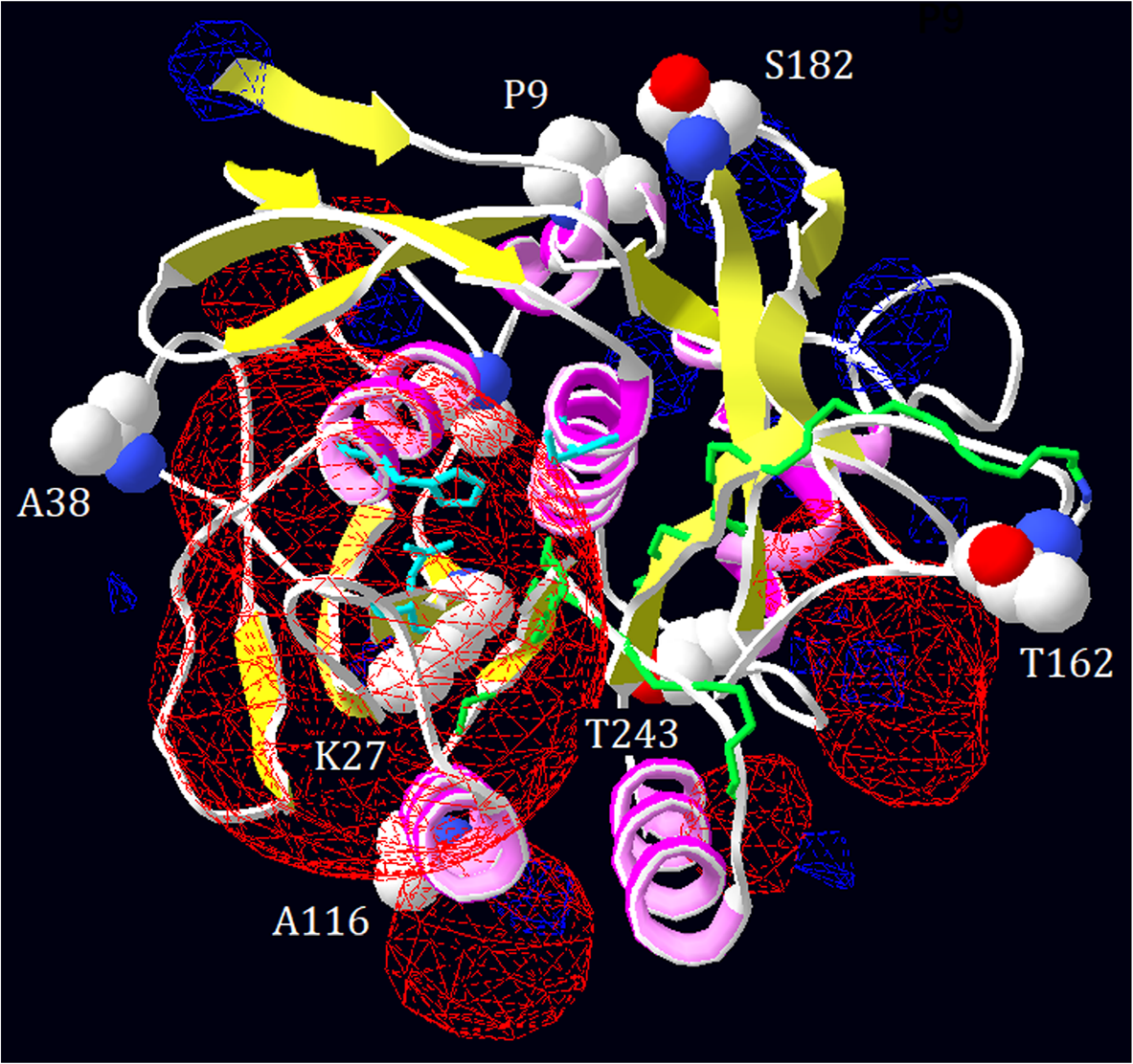
Structural modeling of the alkaline protease DHAP. The electrostatic potential was computed, and the active sites (D32, H64, and S221 shown as sticks) and the seven substitutive sites (shown as balls) are labeled

Engineering enzymes that can tolerate and maintain high activity at cold temperatures is desirable for various industrial applications, not least because it reduces energy consumption during industrial processes [5, 31]. Significant research efforts have therefore been invested in this area. For example, in silico analyses of the homologous counterparts between mesophilic and psychrophilic proteins have been performed, along with experimental studies including in vitro random mutations as well as site-directed mutagenesis [32, 33]. Previously, several variants of DHAP have been engineered to improve the activity of the enzyme at cold temperatures by site-directed mutagenesis [23, 24]. In this study, through in vitro molecular evolution and extensive screening, we obtained several new variants with improved catalytic activity towards both of the substrates, casein and AAPF-*p*N, especially at lower temperature (15 °C). To interpret the experimental data, homologous structural modeling of DHAP was performed (Fig. 6). The alkaline protease of DHAP is a typical serine protease with a catalytic triad (D32, H64, and S221). This protease catalyzes the hydrolysis of peptide bonds through the formation of an acyl-enzyme intermediate via the oxygen atom of S221, which is in turn hydrolyzed into free acid by nucleophilic attack aided by a general base (H64) that accepts the proton from the nucleophilic OH group of D32 [4]. However, the substitution sites characterized here were far from the active center (Fig. 1). In addition, in this study, multiple amino acid alignments of several other bacterial proteases were constructed (Additional file 4: Figure S2).

**Fig. 7:**
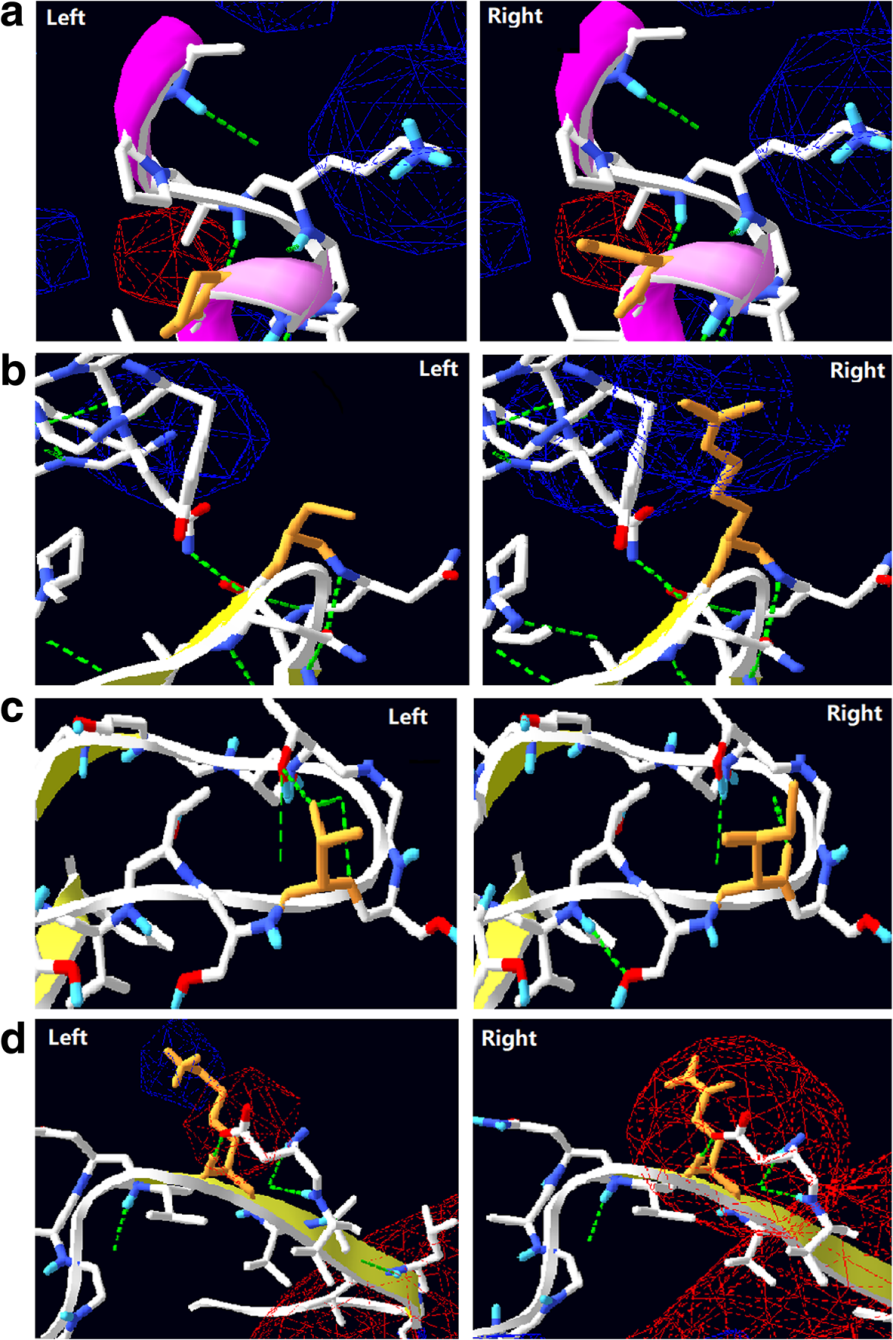
Local view of the substitutions that occurred in the variants of the alkaline protease DHAP. **a**, the substitution of P9 (left) with a serine residue (right); **b**, the substitution of T162 (left) with an isoleucine residue (right); **c**, the substitution of S182 (left) with a lysine residue (right); **d**, the substitution of K27 (right) with a glutamine residue (right)

As shown by structural modeling of DHAP (Fig. 6), the four substitution sites (P9, A38, T162, and S182) were predicated to be exposed on the protein surface. The substitution of P9 with a serine residue led to an increase in catalytic activity towards both the casein and AAPF-*p*N substrates at low temperature (15 °C). The engineered cold-adapted variants of the mesophilic serine protease SSII were reported to contain substitution of the corresponding site (K11) [12]. Furthermore, the proline residue is important in proteins because its side group leads to steric hindrance and increased rigidity [34, 35]. Therefore, substitution of P9 with S9 may lead to more flexibility in this region of the protein (Fig. 7a) [35]. Whereas, conserved proline residues are important for protein stability. For example, substitution of P210 in the subtilisin Carlsberg and P5 in aqualysin I led to a reduction in thermostability [36, 37]. By contrast, substitution of P9 with S9 in DHAP did not result in loss of thermostability (Fig. 4b).

A38, T162, and S182 are localized on various loop regions of DHAP (Fig. 6). These loop regions are thought to confer flexibility in any given protein. Rational loop design, therefore, has been used to increase catalytic activity at low temperature [38]. However, decreased thermostability was often associated with these variants [37, 39]. This phenomenon is recognized as a trade-off between cold activity and thermostability [40]. This situation has been documented for the variant S182R, whose thermostability was decreased in terms of its half-life value (*t*_1/2_). In addition, in silico analysis revealed that the occurrence of lysine residues on the surface of a protein is related to its thermostability [41]. Psychrophilic enzymes tend to have fewer lysine residues exposed on their surface than their mesophilic or thermophilic counterparts, which may stabilize the protein by the formation of salt-bridges or hydrogen bonds [41]. However, substitution of S162 with a lysine residue in DHAP did not enhance its thermostability. This result may be attributed to the lack of formation of a hydrogen bond between the substituted lysine residue in the structural model of DHAP. Instead, this substitution may cause a local change in the electrostatic potential (Fig. 7b), which may affect the flexibility and hence increase the turnover number in terms of catalysis (Table 2).

However, the trade-off between catalytic activity and thermostability does not always occur. For example, substitution of Y195 with a cysteine residue in the alkaline serine protease KP-43 led to an increase in both proteolytic activity and thermostability [42]. The variant T162S achieved a large increase in catalytic activity by more than 2.5-fold with both substrates at 15 °C without loss of thermostability as measured by the t_2/1_ value (Fig. 5b). Based on structural modeling, substitution of T162 with a serine residue led to loss of the H-bond to connect to T159 (Fig. 7c), potentially making the local loop region more flexible. Furthermore, this site is near to the substrate-binding loop, which may therefore mediate access to the catalytic center. In conclusion, the three substitutions A38, T162, and S182 may all contribute to the loop conformation, leading to an increase in the catalytic turnover number.

The other three sites (K27, A116, and T243) were buried within the structural conformation of DHAP, as determined by structural modeling (Fig. 6). K27 is conserved across the mesophilic subtilisin-like protease and localized on the second β-sheet (Fig. 6, Additional file 4: Figure S2). By contrast, the corresponding site in the cold-adapted subtilisin-like protease S41, TA39S, is not occupied by a lysine but instead is occupied by an asparagine residue (Additional file 4: Figure S2). The corresponding site K27 in *B. lentus* subtilisin has been reported to be associated with cold activity [43]. In addition, substitution of K27 with glutamine may change the local electrostatic potential (Fig. 7d). Therefore, substitution of K27 with glutamine, an asparagine-like amino acid, may contribute to an increase in the catalytic activity at low temperature (Fig. 1 and Table 1). A116 is not conserved across all proteases and is localized in the fourth α-helix (Additional file 3: Figure S1 and Fig. 6). Substitution of A116 with threonine does not seem to interfere with the interaction between nearby groups based on structural modeling. Since the α-helix and β-sheet in a protein are usually regarded as more rigid than the loop region, a large sidechain group of threonine may dominate the space, potentially perturbing the local structure. A similar result was also observed for another variant (W106 K) on the same helix, which showed an increase in the catalytic turnover number, without loss of thermostability [24]. T243 is localized on the seventh α-helix, which is buried far behind the catalytic center. Substitution of T243 with serine may strength the helix, potentially improving the thermostability (Fig. 5b).

Finally, four combined variants were constructed from three sites: P9, K27, and T162, and catalytic activity with the macromolecular substrate casein was found to be further increased, especially at low temperature. For example, the variant (P9S/K27Q) showed an increase in caseinolytic activity of more than 5-fold compared with the wt, which was higher than that of the previous variant (W106 K/V149I) [24]. By contrast, the catalytic efficiency of variant P9S/K27Q was lower than that of variant W106 K/V149I for the hydrolysis of AAPF-*p*N. Therefore, the substrate specificity of these variants may be different. When using the synthetic substrate (AAPF-*p*N), the turnover number (*k*_cat_) showed a dramatic increase, especially for the combined variants (P9S/T162I, P9S/K27R, and P9S/K27R/T162I). This result differed from that of other bacterial alkaline protease variants, which exhibited an increase only in caseinolytic activity, but a decrease in the hydrolysis of synthetic peptides [9, 42]. However, the effects of individual substitutions on catalytic activity may be accumulative and can be integrated into a single variant [44].

## Conclusions

Directed evolution and site-directed mutagenesis were combined to engineer variants of an alkaline serine protease from *B. pumilus*. All of the variants exhibited an increase in hydrolytic efficiency at low temperature when both casein and synthetic peptide were used as substrates. The variants (P9S/T62I, P9S/K27R) showed an approximate 5-fold increase in specific activity towards casein and a 2.6-fold enhancement in catalytic efficiency (*k*_cat_/*K*_m_) towards the synthetic peptide at 15 °C compared with the wt enzyme. Meanwhile, none of the variants showed a significant decrease in thermostability. The enhancement of catalytic efficiency is mainly attributed to a significant increase in the turnover number (*k*_cat_). These data suggest that engineering variants with low-temperature activity is not always associated with a loss of thermostability. In fact, the enhancement of cold activity and thermostability can be integrated into a single variant of bacterial alkaline protease.

## Additional files

Additional file 1 **Table S1.** The primers used to construction of DHAP variants by site-directed mutagenesis. (DOCX 16 kb)

Additional file 2 **Table S2.** Summary of the caseinolytic activity of the DHAP variants. (DOCX 19 kb)

Additional file 3 **Figure S1.** SDS-PAGE analysis of the wt and variants of the alkaline protease (DHAP). Lane 1–12 represents the wt, P9S, A38V, A116T, T162I, S182R, T243S, A1G/K27 K, P9S/K27Q, P9S/T162I, K27Q/T162I, and P9S/K27Q/T162I. (PDF 398 kb)

Additional file 4 **Figure S2.** Multiple alignments of the selected bacterial alkaline proteases. DHAP (GI:38373994); Savi (subtilisin Savinase, PDB code: 1NDQ); BgAP (GI:227300899); SAPB (GI: 1869288); SubE (subtilisin E, PDB code: 1SCJ); SubN (subtilisin NOVO, PDB code: 2SBT); Carl (subtilisin Carlsberg, PDB code: 1SBC); WF146 (GI:34851156); S41 (psychrophilic subtilisin-like protease, PDB code: 2GKO); TA39 (psychrophilic subtilisinlike protease, GI: 40201). “s” represents beta sheet; “h” represents a helix. The substitution sites in red color are labeled. (PDF 340 kb)

## Abbreviations

AAPF-*p*N: *N*-succinyl-*L*-Ala-Ala-Pro-Phe-*p*-nitroanilide
Cm: Chloramphenicol
Kan: kanamycin
LB: Luria–Bertani
wt: wild-type
