# Supplementary material for: Engineering Bacillus pumilus alkaline serine protease to increase its low-temperature proteolytic activity by directed evolution
Source: BMC Biotechnol. 2018 Jun 1;18:34. doi: 10.1186/s12896-018-0451-0 (PMC5984802; doi:10.1186/s12896-018-0451-0)
Supplement: Supplementary file 1 — Table S1. The primers used to construction of DHAP variants by site-directed mutagenesis. (DOCX 16 kb) [file 12896_2018_451_MOESM1_ESM.docx]

**Table S1.** The primers used to construction of DHAP variants

by site-directed mutagenesis*

| **Primer** | **Length** | **Sequence (5’-3’)** |
| --- | --- | --- |
| T290I-F | 30 | GGCTCT***ATC***AGCACCGTAGGCTATCCTGCA |
| T291I-R | 30 | GGTGCT***GAT***AGAGCCAGTGGAACCTGAATT |
| K135Q-F | 30 | AACGTC***CAA***GTAGCTGTCCTTGATACTGGA |
| K135Q-R | 30 | AGCTAC***TTG***GACGTTAGCACCTTTATAACC |
| P117S-F | 30 | GGTATC***TCT***CAAATCAAAGCTCCAGCCGTA |
| P117S-R | 30 | GATTTG***AGA***GATACCATAAGGGACGGTTTG |

*: the letters in the primer sequence represent the mutation site.
