# Supplementary material for: Engineering Bacillus pumilus alkaline serine protease to increase its low-temperature proteolytic activity by directed evolution
Source: BMC Biotechnol. 2018 Jun 1;18:34. doi: 10.1186/s12896-018-0451-0 (PMC5984802; doi:10.1186/s12896-018-0451-0)
Supplement: Supplementary file 2 — Table S2. Summary of the caseinolytic activity of the DHAP variants. (DOCX 19 kb) [file 12896_2018_451_MOESM2_ESM.docx]

**Table S2.** Summary of the caseinolytic activity of the DHAP variants*

| **Variants** | **Caseinolytic activity (U/min•mg)** | | **Variants** | **Caseinolytic activity (U/min•mg)** | |
| --- | --- | --- | --- | --- | --- |
|  | 15°C | 50°C |  | 15°C | 50°C |
| **E-25G** | 441 ±2% | 2,144 ±6% | **S182R** | 739 ±17% | 11,128 ±5% |
| **E-25K** | 615 ±10% | 7,925 ±4% | **A231V** | 727 ±6% | 13,989 ±7% |
| **A-23G** | 647 ±7% | 6,651 ±11% | **N240S** | 458 ±4% | 9,720 ±5% |
| **P9S** | 731 ±17% | 5,152 ±15% | **T243S** | 837 ±6% | 7,664 ±2% |
| **A15V** | 751 ±11% | 5,339 ±27% | **A254V** | 432 ± 3% | 5,955 ±10% |
| **A18T** | 486 ±10% | 3,837 ±2% | **N-45D/A74G** | 485 ±9% | 3,976 ±16% |
| **K22N** | 432 ±8% | 5,096 ±14% | **K-39/P14T** | 561 ±26% | 5,136 ±9% |
| **A38V** | 403 ±11% | 2,371 ±6% | **Y-26P/N144D** | 595 ±11% | 3,304 ±16% |
| **A48V** | 561 ±17% | 5,181 ±10% | **E-3G/A88V** | 440 ±10% | 5,619 ±4% |
| **F61S** | 795 ±19% | 6,389 ±9% | **A1G/K27Q** | 938 ±13% | 5,840 ±12% |
| **T78A** | 495 ±11% | 5,968 ±2% | **V16A/A228T** | 569 ±5% | 3,389 ±18% |
| **A85V** | 580 ±23% | 9,411 ±6% | **A24V/V115I** | 432 ±3% | 4,147 ±7% |
| **A116T** | 1,065 ±13% | 9,808 ±10% | **A242T/T243A** | 411 ±3% | 7,333 ±5% |
| **N118D** | 855 ±11% | 3,499 ±8% | **T-56A/A38V/I205V** | 381 ±5% | 7,840 ±5% |
| **D120N** | 485 ±8% | 9,680 ±10% | **V-48A/A116S/G131S** | 517 ±16% | 5,715 ±12% |
| **G131D** | 429 ±12% | 4,949 ±3% | **T-28M/V37A/A38V** | 859 ±7% | 10,621 ±4% |
| **T133I** | 529 ±11% | 4,893 ±14% | **T78A/V185A/N240Y** | 450 ±8% | 2,677 ±11% |
| **T162A** | 600 ±11% | 5,107 ±14% |  |  |  |
| **T162I** | 630 ±3% | 12,888 ±9% | **WT** | 387 ±5% | 4,597 ±9% |

*: the signal “-” means the order of amino acid residue opposite to the first residue of the mature peptide of alkaline protease DHAP.
