# Supplementary material for: Engineering Bacillus pumilus alkaline serine protease to increase its low-temperature proteolytic activity by directed evolution
Source: BMC Biotechnol. 2018 Jun 1;18:34. doi: 10.1186/s12896-018-0451-0 (PMC5984802; doi:10.1186/s12896-018-0451-0)
Supplement: Supplementary file 3 — Figure S1. SDS-PAGE analysis of the wt and variants of the alkaline protease (DHAP). Lane 1–12 represents the wt, P9S, A38V, A116T, T162I, S182R, T243S, A1G/K27 K, P9S/K27Q, P9S/T162I, K27Q/T162I, and P9S/K27Q/T162I. (PDF 398 kb) [file 12896_2018_451_MOESM3_ESM.pdf]

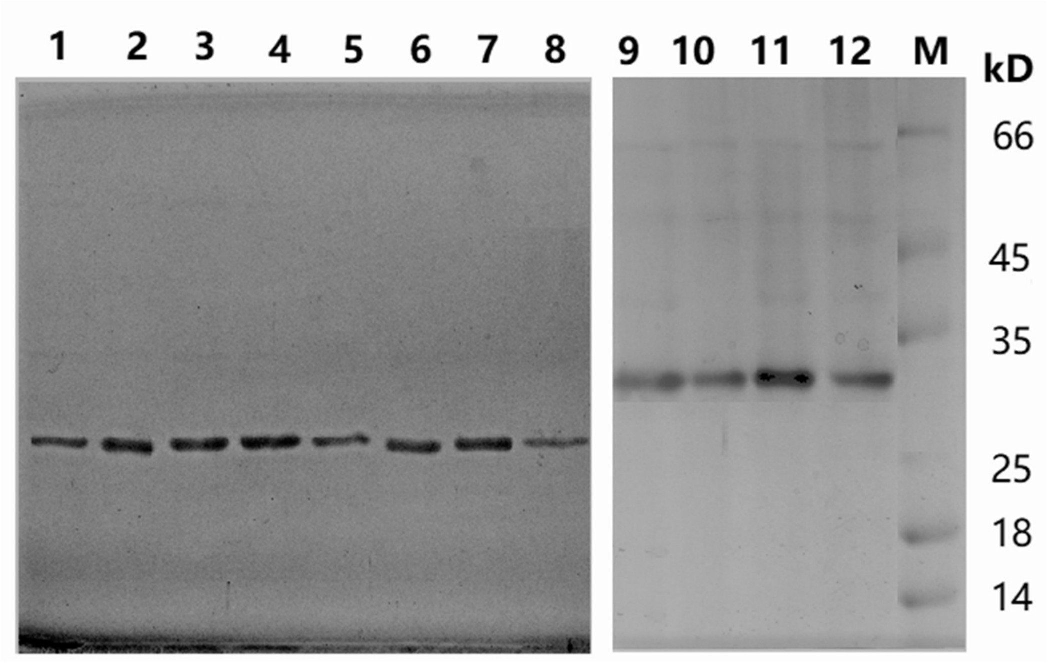

Fig. S1. SDS-PAGE analysis of the wt and variants of the alkaline protease (DHAP). Lane 1-12 represents the wt, P9S, A38V, A116T, T162I, S182R, T243S, A1G/K27K, P9S/K27Q, P9S/T162I, K27Q/T162I, and P9S/K27Q/T162I.
