# Supplementary material for: Engineering Bacillus pumilus alkaline serine protease to increase its low-temperature proteolytic activity by directed evolution
Source: BMC Biotechnol. 2018 Jun 1;18:34. doi: 10.1186/s12896-018-0451-0 (PMC5984802; doi:10.1186/s12896-018-0451-0)
Supplement: Supplementary file 4 — Figure S2. Multiple alignments of the selected bacterial alkaline proteases. DHAP (GI:38373994); Savi (subtilisin Savinase, PDB code: 1NDQ); BgAP (GI:227300899); SAPB (GI: 1869288); SubE (subtilisin E, PDB code: 1SCJ); SubN (subtilisin NOVO, PDB code: 2SBT); Carl (subtilisin Carlsberg, PDB code: 1SBC); WF146 (GI:34851156); S41 (psychrophilic subtilisin-like protease, PDB code: 2GKO); TA39 (psychrophilic subtilisinlike protease, GI: 40201). “s” represents beta sheet; “h” represents a helix. The substitution sites in red color are labeled. (PDF 340 kb) [file 12896_2018_451_MOESM4_ESM.pdf]

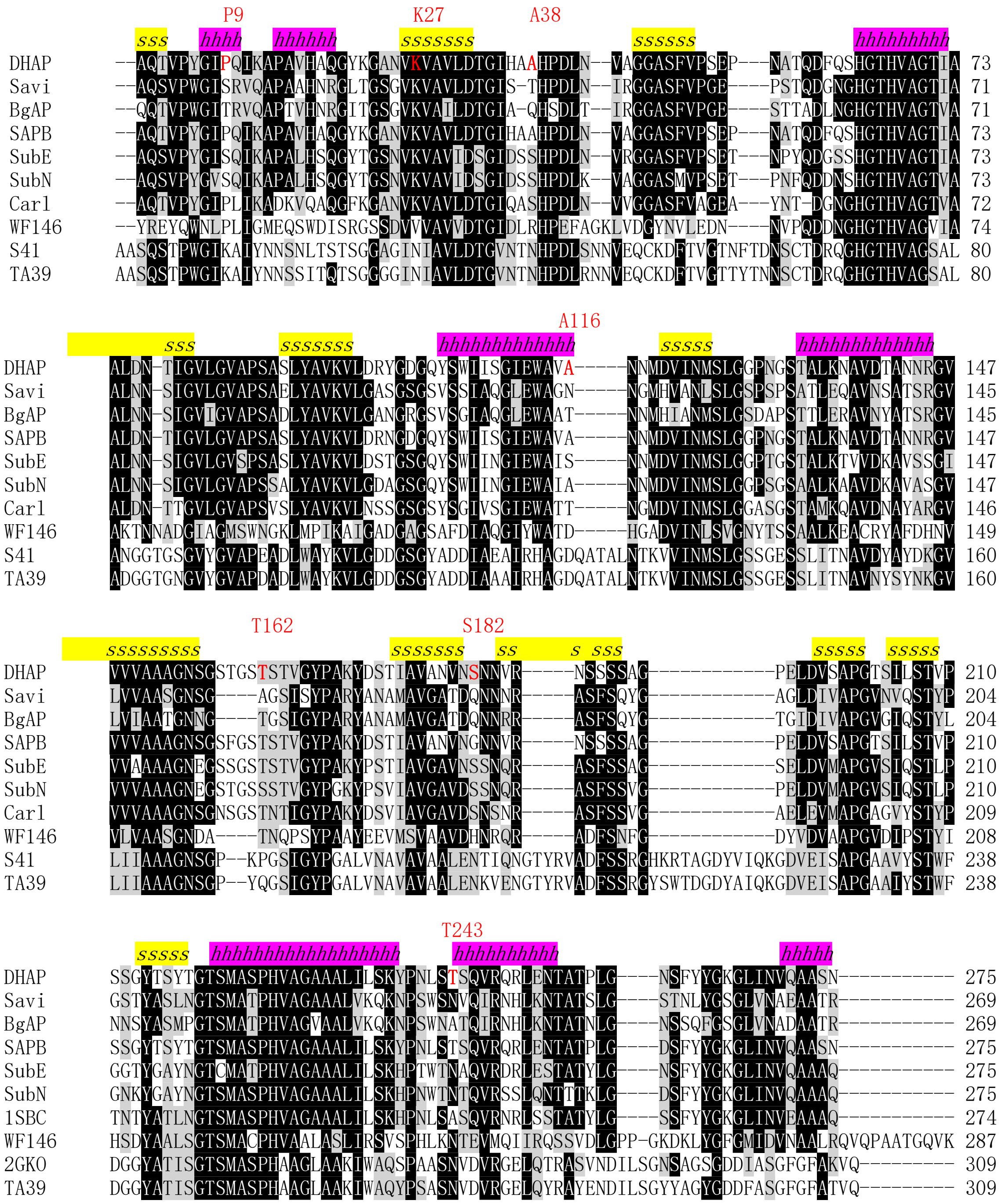

Fig. S2. Multiple alignments of the selected bacterial alkaline proteases. DHAP (GI:38373994); Savi (subtilisin Savinase, PDB code: 1NDQ); BgAP (GI:227300899); SAPB (GI: 1869288); SubE (subtilisin E, PDB code: 1SCJ); SubN (subtilisin NOVO, PDB code: 2SBT); Carl (subtilisin Carlsberg, PDB code: 1SBC); WF146 (GI:34851156); S41 (psychrophilic subtilisin-like protease, PDB code: 2GKO); TA39 (psychrophilic subtilisin-like protease, GI: 40201). "*s*" represents beta sheet; "*h*" represents a helix. The substitution sites in red color are labeled.
